# Case Report: ALK rearranged locally advanced lung adenocarcinoma showing inconsistent radiographic findings and pathological responses during neoadjuvant alectinib therapy

**DOI:** 10.3389/fphar.2023.1140894

**Published:** 2023-08-17

**Authors:** Peijun Cao, Qingchun Zhao, Yongwen Li, Ruifeng Shi, Guangsheng Zhu, Zihe Zhang, Hongbing Zhang, Minghui Liu, Sen Wei, Hongyu Liu, Jun Chen

**Affiliations:** ^1^ Department of Lung Cancer Surgery, Tianjin Medical University General Hospital, Tianjin, China; ^2^ Tianjin Lung Cancer Institute, Tianjin Key Laboratory of Lung Cancer Metastasis and Tumor Microenvironment, Tianjin Medical University General Hospital, Tianjin, China

**Keywords:** EML4-ALK fusion gene, pulmonary adenocarcinoma, neoadjuvant therapy, alectinib, pseudoresistance

## Abstract

Alectinib has been approved as first-line treatment for anaplastic lymphoma kinase (ALK)-positive non-small cell lung carcinoma. Oncologists are also exploring the possibility of applying alectinib in the perioperative period. Here, we present a patient with locally advanced lung adenocarcinoma associated with *EML4*-*ALK* fusion mutation, who received neoadjuvant chemotherapy and alectinib treatment, and then underwent thoracoscopic left lower lung lobectomy. The patient initially received eight chemotherapy cycles and achieved partial remission. After eight cycles of chemotherapy, the lymph nodes in the hilar region again enlarged. The patient was then switched to 4 months of alectinib therapy, but no significant lesion changes were detected on imaging during this period. This raised the question of whether the patient developed alectinib resistance. The pathological findings of the postoperative lung lobe specimens indicated extensive necrosis in the tumor area with no residual tumor cells and massive chronic inflammatory cell infiltration around the tumor area, confirming inconsistency between the imaging findings and pathological results. Multi-point tumor specimen sampling was postoperatively performed. Tumor immune-related gene expression was detected in the sample with the help of the PanCancer IO360™ panel based on the nCounter platform. This is a rare case of a patient who was treated with neoadjuvant alectinib and had paradoxical radiographic findings and pathological responses. The possibility that intratumoral immune heterogeneity was responsible for this phenomenon has been discussed. Based on the findings, it is argued that the pathological response should be an important basis for assessing the effectiveness of neoadjuvant alectinib therapy.

## Introduction

Lung cancer has received widespread attention owing to its high incidence and mortality rate. The lung cancer treatment paradigm has changed with the development of genetic testing and molecular targeted therapy. Currently, anaplastic lymphoma kinase (ALK) inhibitors are used as the standard first-line treatment for ALK fusion-positive advanced non-small cell lung carcinoma (NSCLC), and alectinib is a second-generation ALK inhibitor. According to the global Alex research update, the average progression-free survival (PFS) duration with alectinib treatment was 35 months, and the treatment greatly improved patient survival time (Mok et al., 2020). Today, there is a growing interest in the possibility of treating patients with alectinib at an earlier stage, including preoperative neoadjuvant or postoperative adjuvant therapy in operable patients. In previous retrospective studies with small sample sizes, patients treated with neoadjuvant alectinib achieved partial remission (PR) without serious side effects (Zhang et al., 2020; Gu et al., 2021; Yue et al., 2021). This finding suggests that alectinib treatment has good efficacy and safety; however, the outcomes are temporarily unsupported by data from large clinical trials.

Patients’ therapeutic responses are usually evaluated according to lesion changes by oncologists or surgeons, using radiographic techniques such as computed tomography (CT). However, it is known that imaging findings can sometimes mislead doctors. For example, approximately 1.8%–6.9% of patients with NSCLC receiving immunotherapy will experience pseudoprogression, a phenomenon in which an initial increase in tumor size or appearance of new lesions followed by remission of enlarged lesions is observed on imaging; however, the enlarged lesions have been verified and shown to consist mainly of hemorrhage, necrosis, edema, and immune cell infiltration by pathologists (Zhou et al., 2020). Pseudoprogression in NSCLC can occur naturally during immunotherapy and has also been reported in patients treated with alectinib for central nervous system (CNS) metastases, where there is a CNS radionecrosis risk, which can manifest as pseudoprogression on imaging (Ou et al., 2015; Ou et al., 2016).

Here, we present a patient with NSCLC, who showed inconsistent radiographic findings and pathological responses during neoadjuvant alectinib therapy. Next-generation techniques revealed intratumor heterogeneity at the immune landscape level.

### Case presentation

**FIGURE 1:**
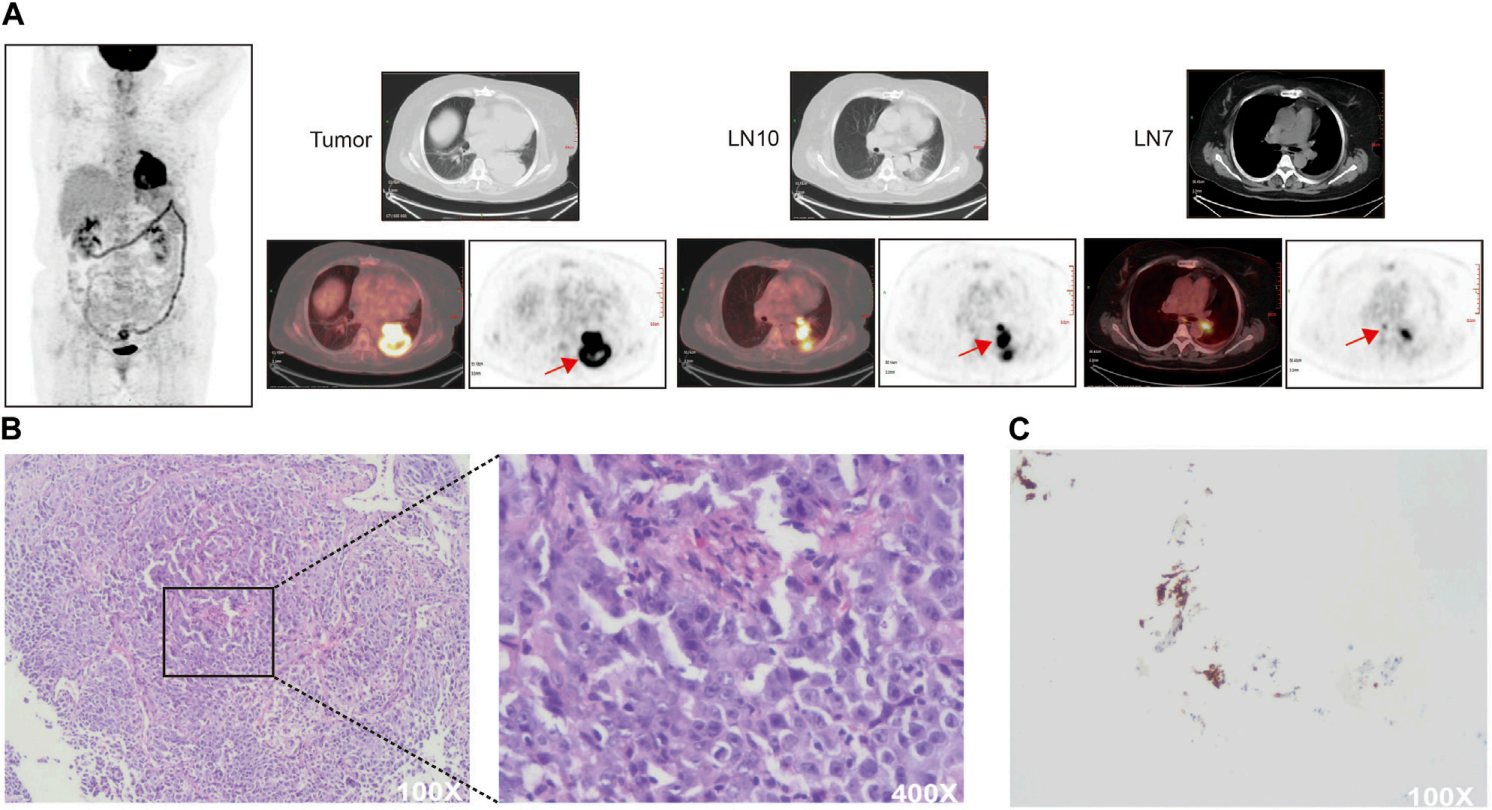
**(A)** Baseline PET-CT scan of the patient. SUVmax (LN10) = 17.3, SUVmax (LN7) = 5.6 **(B)** Hematoxylin and eosin staining of pretreatment specimens. **(C)** Immunohistochemical detection of ALK protein expression in pretreatment specimens. Tissue paraffin sections were blocked and incubated with primary antibody against ALK (1:50, DAKO North America, United States) overnight at 4°C. PBS was substituted for the antibody as a negative control. Cytoplasmic staining was considered ALK positive.

A 55-year-old woman who never smoked was admitted to the hospital on 1 January 2019, with the main complaints of coughing and sputum, wheezing for 6 months, and left-sided back pain for 3 days. The patient had 10-year chronic obstructive pulmonary disease and 7-year hypertension. She had undergone thymectomy 13 years previously and has been taking pyridostigmine bromide plus atropine. Her mother is now deceased but had pulmonary fibrosis and esophageal cancer. The rest of her family members have no malignancy history. On admission, chest CT showed an enlarged left lower lung hilum with an irregular 75.9-mm-diameter soft tissue mass in the left lower lobe. Multiple lymph nodes in the left lung hilum and mediastinum appeared partially enlarged. Tumor marker levels in the serum were significantly elevated (carcinoembryonic antigen (CEA) = 108.18 ng/mL, neuron-specific enolase (NSE) = 30.82 μg/L). Positron emission tomography-computed tomography (PET-CT) and brain magnetic resonance imaging showed no signs of distant metastases (Figure 1A). Fiberoptic bronchoscopy revealed narrowing of the opening in the dorsal segment of the left lower lobe and an obstructing mass in the opening of the basal segment. A biopsy of the mass in the left lower lung basal segmental opening was performed under bronchoscopy, and the pathological findings indicated lung adenocarcinoma (Figure 1B). The carcinoma was clinically staged as stage IIIB (cT4N2M0), and there was no surgery indication. Next-generation sequencing of specimens obtained by bronchoscopy identified an *EML4*-*ALK* fusion mutation which is inherently a translocation fusion caused by inversion within the chromosome arm, and subsequently, an immunohistochemistry test indicated high expression of the ALK protein in the tumor tissue samples of this patient (Figure 1C).

**FIGURE 2:**
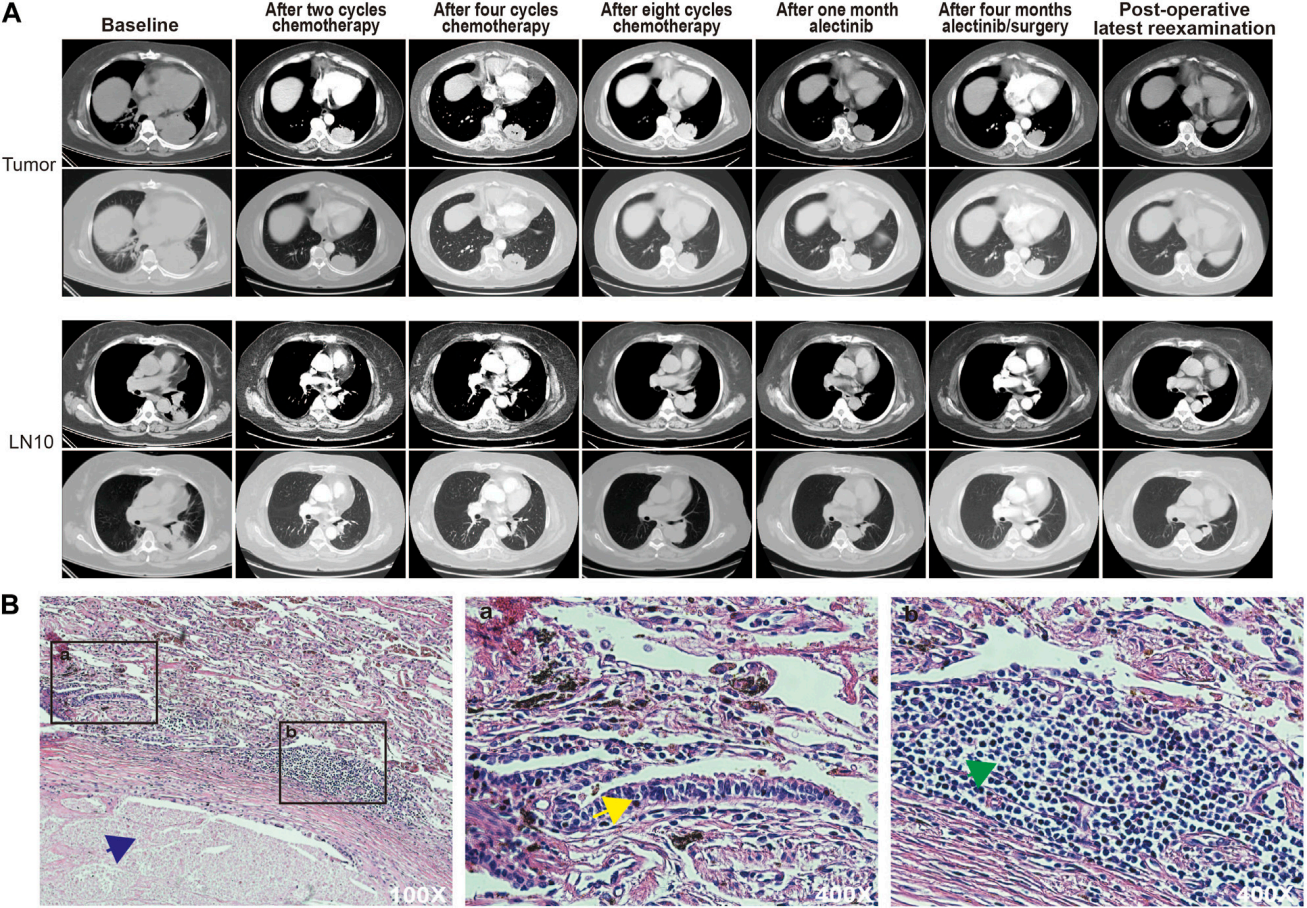
**(A)** CT scans of the patient at each stage of treatment. **(B)** Hematoxylin and eosin staining of the treated specimen. Blue arrows point to necrotic areas, yellow arrows point to atypical hyperplastic cells, and green arrows point to infiltrating lymphocytes.

The patient opted for chemotherapy with pemetrexed 1.0 g, nedaplatin 155 mg, and apatinib 250 mg when the genetic test results were not clear, due to the patient’s eagerness to get treatment. CT showed that the lesion was 44.7 mm in diameter after eight chemotherapy cycles (a reduction of 41.11% compared to the diameter in the pretreatment period), and the findings indicated that she had achieved PR according to the Response Evaluation Criteria in Solid Tumors (RECIST) version 1.1. Although the patient’s subcarinal region lymph nodes (LN7) did not change significantly during treatment (Supplementary Figure S1A), the patient’s hilar region lymph nodes (LN10) became large again at the end of eight cycles of chemotherapy (Figure 2A, Supplementary Figure S1B). After this point, she started taking alectinib 600 mg orally twice a day in the pursuit of further antitumor effects. CT showed that the lesion diameter was 42.4 mm 4 months later, with no significant change from the diameter at the end of chemotherapy (Figure 2A). However, at this time, serum levels of tumor markers, especially CEA, had fallen to lower levels (Supplementary Figure S1C). A thoracoscopic left lower lobe resection was performed on 19 October 2020, after obtaining consent from the patient and her family. Mediastinal lymph node dissection was not successfully performed because of the high bleeding risk due to severe lymph node tissue adhesions and her inability to tolerate a prolonged operation due to poor lung ventilation. The pathological findings of the postoperative lung lobe specimens indicated extensive necrosis in the tumor area with no residual tumor cells and massive chronic inflammatory cell infiltration around the tumor area, confirming the preoperative effectiveness of alectinib (Figure 2B).

**FIGURE 3:**
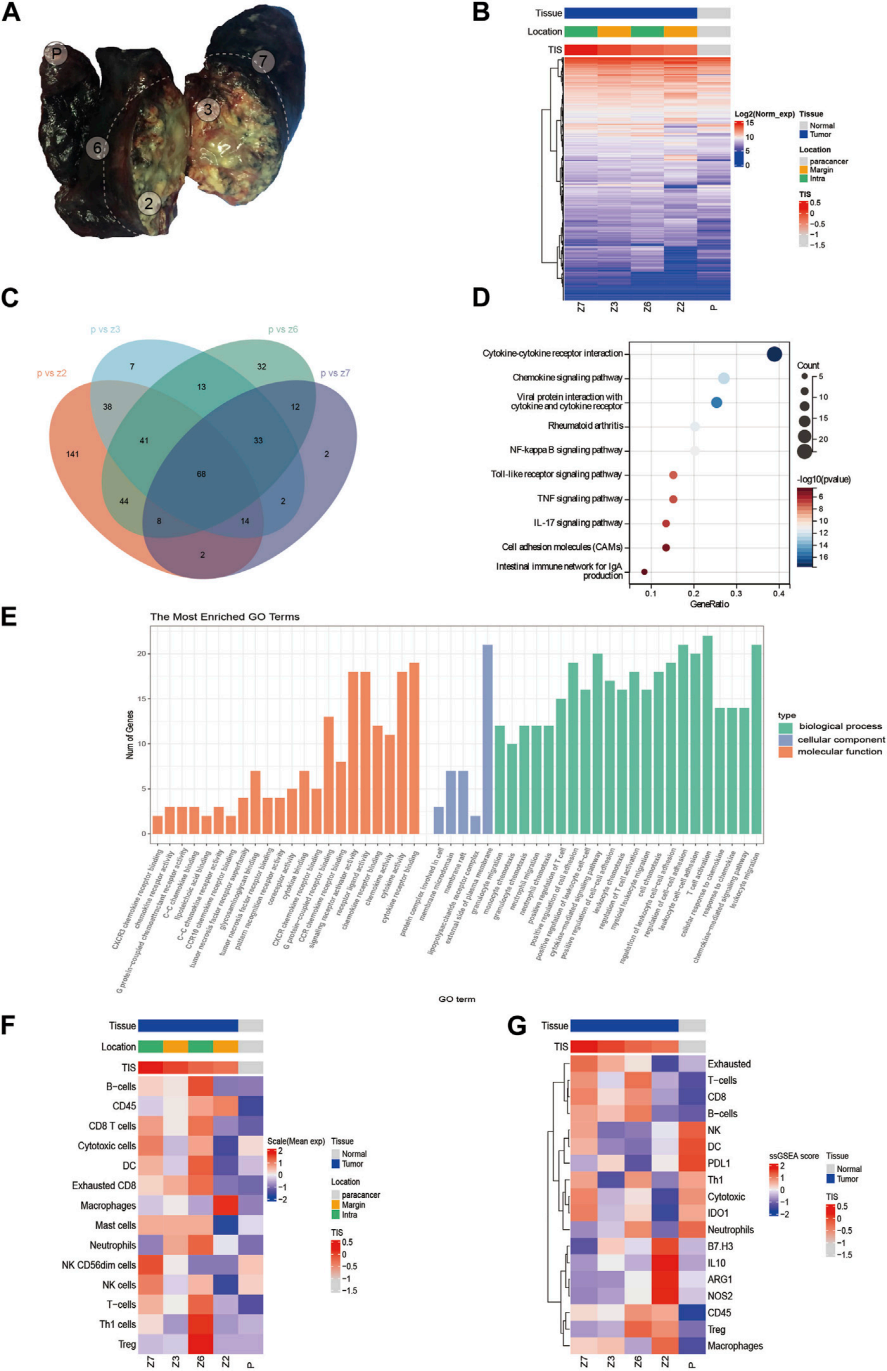
**(A)** Sampling point location. **(B)** Clustering analysis of 770 gene expressions. **(C)** Venn diagram showing differentially expressed genes (DEGs) common to each tumor tissue sampling site and paracancerous tissue. **(D)** Kyoto Encyclopedia of Genes and Genomes (KEGG) analysis of DEGs. **(E)** Gene Ontology (GO) analysis of DEGs. **(F)** Evaluation of immune cell infiltration. **(G)** Heat map of the expression of immune-related signatures.

Multi-point tumor specimen sampling was performed (Figure 3A) with consent from the patient and her family. Z2 and Z3 were closer to the visceral pleura and located at the lung margins, and were classified as the margin group in later analysis, while Z6 and Z7 were closer to the internal lung tissue and were classified as the intra group in later analysis. Given the extensive necrotic tissue in the tumor samples, we selected the PanCancer IO360™ panel, which is based on the nCounter platform (NanoString Technologies, Seattle, WA, United States) with a high tolerance for sample quality, to detect the expression of 770 genes related to tumor immunity in the samples. Hierarchical clustering analysis was performed. We found that there was no significant clustering at the four sampling sites, and some differences were seen between sampling locations (Figure 3B). Further, we identified differentially expressed genes (DEGs) between each tumor tissue sampling site and paracancer normal tissue (Figure 3C), and the 68 DEGs common to each combination were subjected to Gene Ontology (GO) enrichment analysis and Kyoto Encyclopedia of Genes and Genomes (KEGG) pathway analysis. The KEGG analysis showed that the DEGs were mainly enriched in signaling pathways such as cytokine-cytokine receptor interaction and the chemokine signaling pathway (Figure 3D). GO analysis showed that DEGs were mainly related to granulocyte migration, monocyte chemotaxis, granulocyte chemotaxis, neutrophil migration, neutrophil chemotaxis, and positive regulation of T cells (Figure 3E).

Tumor inflammation signature (TIS) has been jointly developed by Nanostring and Merck & Co., and is an immune microenvironment gene expression profile marker. The biomarker includes 18 genes that are highly correlated with clinical response to immune checkpoint inhibitors. These include the interferon-γ (IFN-γ) signaling pathway, and T cell and NK cell abundance. Currently, TIS has made its mark in several clinical trials for tumor immunotherapy (Turan et al., 2018). TIS scoring of four tumor sampling sites and paracancer samples revealed that the TIS scores for tumor tissues were significantly higher than the score for paracancer tissue. The scores for the four tumor areas were 0.182 (Z7), −0.004 (Z3), −0.228 (Z6), and −0.313 (Z2), while the score for paracancer tissue was −1.082. Furthermore, the scores for the intra group were higher than those for the margin group, with Z7 having the highest TIS score and Z2 having the lowest TIS-related gene expression (Supplementary Figure S1D). Moreover, immune cell infiltration was assessed in each sample. The results showed that Z6 tissues had the highest infiltration percentage of B cells and regulatory T cells (Treg), Z6 and Z7 tissues had higher percentages of CD8+ T cells and DCs, and Z2 tissues had the highest percentage of macrophages (Figure 3F). Our patient additionally had low expression levels of immune-related negative indicators, such as B7-H3, IL10, Treg, ARG1, and NOS2 (Figure 3G). The expression levels of immune checkpoint molecules at each sampling were explored further. The expression level of cytotoxic T lymphocyte antigen 4 (CTLA-4) was the highest in Z6 compared with the other samples, and the expression levels of several immune checkpoint markers (PDL2, TIM3, and 4-1BB) were higher in Z2 than in the other samples (Supplementary Figure S1E).

The patient is currently being treated with adjuvant alectinib and no tumor recurrence or metastasis was found at follow-up until January 2023. In addition, adjuvant alectinib treatment did not cause significant discomfort to the patient.

## Discussion

Different NSCLC patients often respond differently to the same treatment modality owing to tumor heterogeneity. Among 11 ALK-positive N2 patients reported by Wu Yilong, 10 had PR after neoadjuvant therapy with crizotinib, 1 had SD, 2 achieved pathological complete remission, 6 experienced disease recurrence, and 5 received first-line treatment with crizotinib and achieved a longer sustained remission duration (Zhang et al., 2019). Neoadjuvant crizotinib treatment down-staged the tumor from N2 to N0 in one of the patients assessed as having SD, although the tumor had only decreased in size by 10.7%. As reported by Yi and Zhong et al., two patients with ALK-positive lung adenocarcinoma treated with two neoadjuvant alectinib cycles achieved PR (Zhang et al., 2020; Yue et al., 2021). However, only the patient reported by Zhong et al. achieved major pathological response (MPR) after pathological evaluation. These studies reflect the effectiveness of neoadjuvant ALK-TKI therapy in some way, although the sample sizes of these studies were small. The safety of neoadjuvant targeted therapy and its impact on surgical treatment are of major concern. Considering this case and the studies mentioned previously, no serious adverse effects or postoperative complications from alectinib were found. However, severe lymph node adhesions intraoperatively were found in both this study and the study by Yue et al. (2021). Therefore, whether neoadjuvant alectinib affects lung cancer surgery difficulty and R0 resection rates needs to be explored further. The ongoing phase 2 ALNEO trial is designed to assess neoadjuvant alectinib treatment activity in potentially resectable stage III ALK-positive NSCLC (Leonetti et al., 2021). It is expected that this prospective trial will provide additional insights into neoadjuvant alectinib treatment.

ALK-positive patients treated with first-line alectinib have approximately 35 months of PFS, although there is no clear OS, which suggests that such patients are less likely to develop acquired resistance to alectinib in the short term. Makimoto et al. (2019) reported a case of an ALK-positive lung adenocarcinoma patient who developed resistance to alectinib after only 3 months and was subsequently switched to other ALK inhibitors (ceritinib and crizotinib) with similarly low efficacy. This undoubtedly is a wake-up call that even ALK-positive patients might develop resistance to ALK inhibitors in the short term. In previous studies, shrinkage of the tumor and lymph nodes was observed on imaging after two neoadjuvant ALK-TKI cycles (Zhang et al., 2019; Yue et al., 2021), whereas in this report, preoperative alectinib treatment failed to induce tumor changes on CT, making it difficult to rule out the possibility of the development of short-term resistance. Fortunately, pathological evaluation of postoperative tumor samples allowed us to clarify the complete clearance of tumor cells after neoadjuvant alectinib treatment, and despite the lack of pathological findings of the lymph nodes, considering the radiological findings of the patient’s postoperative follow-up, we strongly believe that the patient achieved complete pathological remission. We therefore argue that pseudoresistance can perhaps contribute to the inconsistency between the radiographic findings and pathological responses. Moreover, pathological results of postoperative specimens revealed that there was abundant immune cell infiltration within the tumor compared with the paratumor tissue after alectinib treatment, which may have caused this pseudoresistance.

In this case, tumor immune-related gene expression was examined in postoperative resected tumor specimens by sampling multiple points with the help of the nCounter-based PanCancer IO360™ panel, with the hope to explore the molecular changes beyond the radiological and pathological presentations in the patient. Subsequently, the TIS results between tumor tissues and paracancerous tissues, and the KEGG and GO analysis of DEGs suggested a strong inflammatory response in tumor tissues. However, there was a different tumor immune microenvironment among the four sampling sites, suggesting a high degree of immune heterogeneity within the tumor. Z2 was infiltrated by fewer types of immune cells close to the necrotic area but had the highest proportion of macrophages. Z6 and Z7 had a richer immune cell infiltration close to the intralung tissue, with a higher proportion of both exhausted CD8+ T cells and immunosuppressive cells (Treg), suggesting an immunosuppressive tumor microenvironment. Treg cells can release inhibitory factors, such as transforming growth factor-β (TGF-β) and interleukin-10 (IL-10), to inhibit effector T cells (Teff) function, while exhausted CD8+ T cells can reduce the production of effector factors, such as IL-2, TNF-α, and IFN-γ, in the face of continuous antigenic stimulation, resulting in progressive loss of function (Shimizu et al., 2018; Jiang et al., 2020). Immune checkpoint inhibitors (ICI) targeting programmed cell death-1 (PD-1), programmed cell death ligand-1 (PD-L1), and CTLA-4 are currently effective treatments for NSCLC. However, these therapies are less effective in those patients who harbor oncogenic driver mutations, such as those involving EGFR or ALK (Gainor et al., 2016; Liu et al., 2018). In a study of 715 patients with non-small cell lung cancer, patients with ALK rearrangements were found to have low PD-L1 expression, low CD8^+^ tumor infiltrating lymphocytes (TIL), and low ICI response rates (Liu et al., 2018). Although the exact mechanism behind this phenomenon has not been fully elucidated, it has been shown that in NSCLC, EML4-ALK fusion generates an immunosuppressive tumor microenvironment (TME) by activating downstream oncogenic signaling pathways (PI3K, MAPK and Hippo pathways) (Ota et al., 2015).

In conclusion, we reported pseudoresistance in an advanced ALK-positive lung adenocarcinoma patient treated with neoadjuvant alectinib. The pathological findings of the postoperative specimens showed extensive necrosis in the tumor area with no residual tumor cells. The tumor-infiltrating immune cell profile of this patient was detected, revealing high intratumoral immune heterogeneity. Through this study, we concluded that pathologic response should be an important basis for assessing the effect of neoadjuvant alectinib treatment.

## Data Availability

Relevant data have been uploaded to the OMIX database. OMIX ID: OMIX002974. Can be downloaded through the following link: https://download.cncb.ac.cn/OMIX/OMIX002974/.
