# Supplementary figures and images for: Case Report: ALK rearranged locally advanced lung adenocarcinoma showing inconsistent radiographic findings and pathological responses during neoadjuvant alectinib therapy
Source: Front Pharmacol. 2023 Aug 17;14:1140894. doi: 10.3389/fphar.2023.1140894 (PMC10470069; doi:10.3389/fphar.2023.1140894)

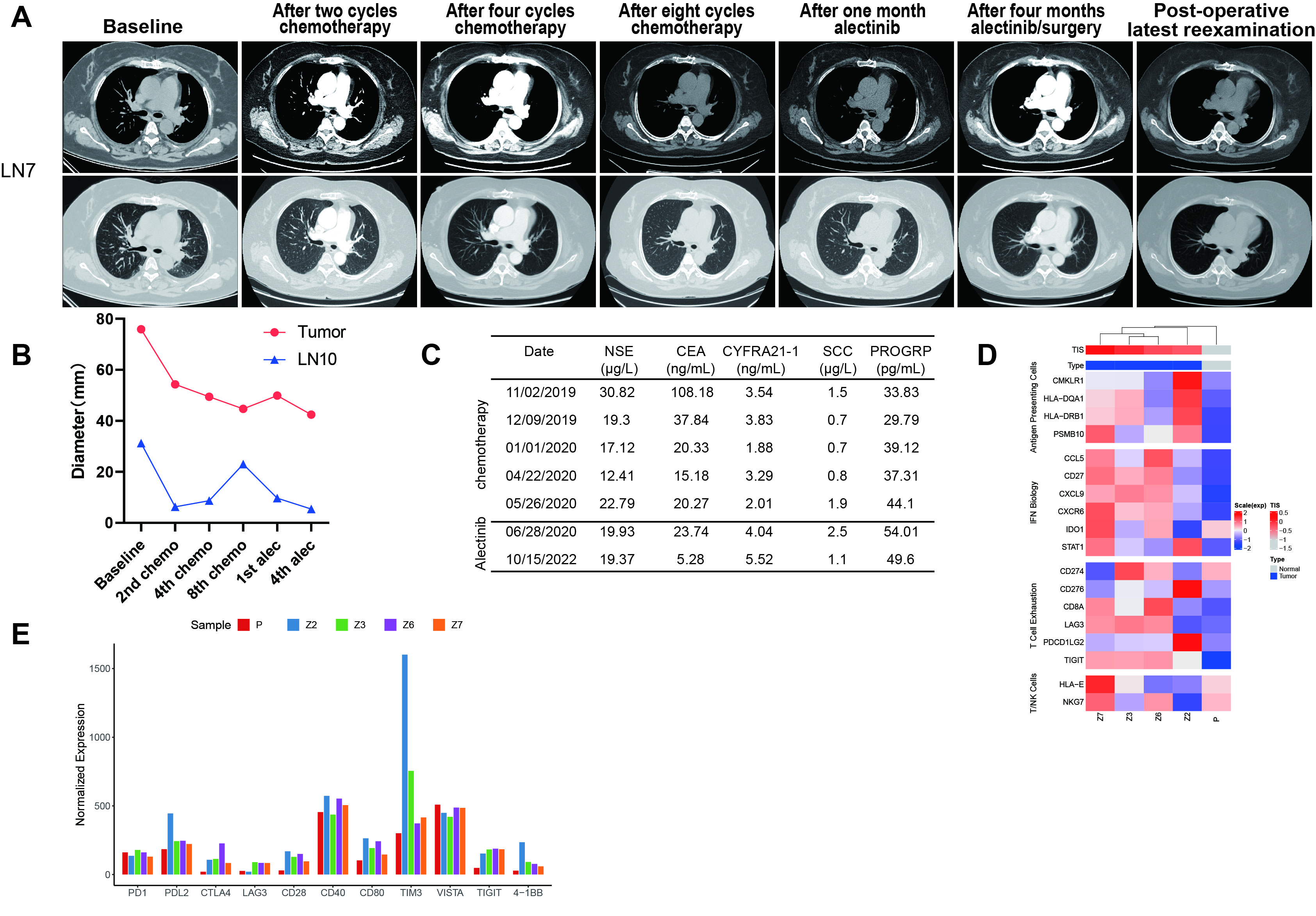

Supplement: Supplementary file 1 [file Image1.JPEG]
